# Development and Validation of a Logic Model for Utilization of Nutrition Support among Patients with Cancer

**DOI:** 10.1155/2020/4513719

**Published:** 2020-06-24

**Authors:** Ngou In Pang, Ruixue Bie, Carolina Oi Lam Ung, Hao Hu

**Affiliations:** State Key Laboratory of Quality Research in Chinese Medicine, Institute of Chinese Medical Sciences, University of Macau, Macao, China

## Abstract

Cancer is the leading cause of morbidity and mortality, and about one in six people die from cancer globally. Approximately 20% to 70% of cancer patients are accompanied with malnutrition, and nutrition support plays an important role among cancer patients. However, the utilization of nutrition support is generally irrational in clinical practices and it is affected by multiple factors. Logic models not only present a framework to improve intervention of health care setting but also identify all the elements, pathways, outcomes, and their relationships between systems. This study developed a logic model of nutrition support for cancer patients based on current literature and conducted interview with medical staff in Macao to validate the logic model. In addition, suggestions were given as references to improve the utilization of nutrition support among cancer patients.

## 1. Introduction

Cancer is one of the leading causes of death, and the World Health Organization (WHO) estimated that 9.6 million people die from cancer globally in 2018. Approximately 20% to 70% of cancer patients are accompanied with malnutrition [1–3]. Nutrition support including enteral nutrition (EN) and parenteral nutrition (PN) not only reduces the complications rates and medical cost but also improves the clinical outcomes of cancer patients, such as body mass index (BMI), immune function, and infection [4–7]. Therefore, nutrition support has played an important role for cancer patients.

To improve and standardize the utilization of nutrition support for cancer patients, the European Society for Clinical Nutrition and Metabolism (ESPEN) published two guidelines, “ESPEN guidelines on nutrition in cancer patients” in 2016 and “ESPEN expert group recommendations for action against cancer related malnutrition” in 2017 [8, 9], and the Chinese Society and Parenteral and Enteral Nutrition (CSPEN) published the guideline, “Guidelines on nutrition support in patients with tumor” in 2017 [10]. However, there are gaps between the utilization of nutrition support in clinical and guidelines, such as the choice, routes, and dosages of nutrition support. A study showed that PN was not used in compliance with guidelines in hospital practice [11]. There were regional, departmental, and disease-based differences in the selection of PN versus EN in Chinese hospitals [12]. Many studies showed that PN was the dominant nutritional mode and the utilization of PN should be improved and guided in Chinese hospitals [13, 14]. On the other hand, cancer patients at difference disease stages with different therapies meet different nutrition needs, and the starting point of nutrition support should be well considered. In addition, the cooperation, attitude, and knowledge of participants, such as physicians and nutritionists, involved in nutrition support among cancer patients, are some of the challenges of utilization of nutrition support [15–17].

As utilization of nutrition support among cancer patients is such a complex problem, it is necessary to figure out the elements of nutrition support and its multiple outcomes. Logic model as a graphical representation can be used to construct frameworks to improve intervention and implementation of health care problems [18, 19]. Logic models are used to conceptualize complex systems, as they not only identify all the components and their relationships between systems but also display the pathways between interventions and outcomes [20, 21]. It is useful and timesaving for researchers to construct logic models for making hypothesis as logic models can reduce the time from start of planning to real practice [22]. There are various types of logic models, such as system-based logic model and process-orientated logic model. System-based logic models present the comprehensive perspective of the system and depict the interactions between interventions and its participants and where it takes place within the system [23]. It is useful to understand the relationship between various interventions in the system through system-based logic models.

To our knowledge, there is no study that presented a logic model of nutrition support for cancer patients. Thus, this study is aimed at developing a logic model of nutrition support for cancer patients based on current literature and conducting interview with medical staff in Macao to validate the logic model. In addition, suggestions are given as references to improve the utilization of nutrition support among cancer patients.

## 2. Method

### 2.1. Research Design and Ethics

This study combined systematic review and qualitative interview to develop and validate the logic model.

This study was conducted according to the guidelines laid down in the Declaration of Helsinki, and all procedures involving research study participants were approved by the Ethics Committee of University of Macau (MYRG2018-00012-ICMS).

### 2.2. Collecting Relevant Literature

To collect relevant literature, we conducted literature search in 3 databases, PubMed, ScienceDirect, and Web of Science, using the following search terms: “nutrition” OR “nutritional” OR “artificial nutrition” OR “nutritional intervention” OR“nutrition intervention” OR “nutrition care” OR “nutrition support” OR “enteral nutrition” OR “parenteral nutrition” AND “cancer” OR “tumor” OR “oncology”.

Literature search was limited to English articles with a time span of 5 years (2014 to 2018), and human was the targeted experimental object. The literature was screened by two reviewers. The two reviewers selected the literature based on the title and abstract at the first-round selection. After the first-round selection, the second-round selection was based on the context.

Inclusion criteria were (1) patients with cancer, whether which types of cancer and disease stages, with or without complication and surgery; (2) patients with cancer receiving nutrition support (enteral nutrition, parenteral nutrition, or single/mixed nutritional ingredient); (3) having outcomes related to the patients with cancer and caused by nutrition support directly or indirectly.

There were 30 published literatures [24–53] that provided information about nutrition support and cancer after removal of duplicates and exclusion of irrelevant literature.

### 2.3. Construction of the Logic Model

For the system-based logic model, PICO framework [54, 55] was used to define the components and research questions, including four elements: participants (P), interventions (I), comparisons (C), and outcomes (O) (see Table 1).

After defining the components of the logic model, we added the elements obtained through literature into the system-based logic model.

### 2.4. Validation of Logic Model

To validate the logic model, an interview was conducted in this study. Interviewees were the medical staff who have worked directly in the field of cancer and nutrition at least 1 year in Macao, and three medical staff participated in the interview.

Invitation letter and consent letter were sent to potential interviewees before the interview. At the beginning of the interview, interviewees were told the introduction and research objective about this study and asked for the permission to tape-record the interview. The interview included three parts: overview, practice and barriers, and solutions and suggestions about nutrition support among cancer patients.

## 3. Results

### 3.1. Literature Analysis

A total of 30 literatures were included in this study; 13 of their study design were mixed methods, followed by randomized trial (RT) and randomized controlled trial (RCT) (see Table 2). 12 literatures were published in 2017, and 10 literatures were published in 2015.

According to the PICO framework, the elements of the selected literature included demographic information, cancer types, disease stages, surgery, treatments, participants, nutrition types, nutrition routes, timing, duration, health outcomes, nonhealth outcomes, adverse reaction, and setting (see Table 3).

### 3.2. First Version of the Logic Model

**Figure 1:**
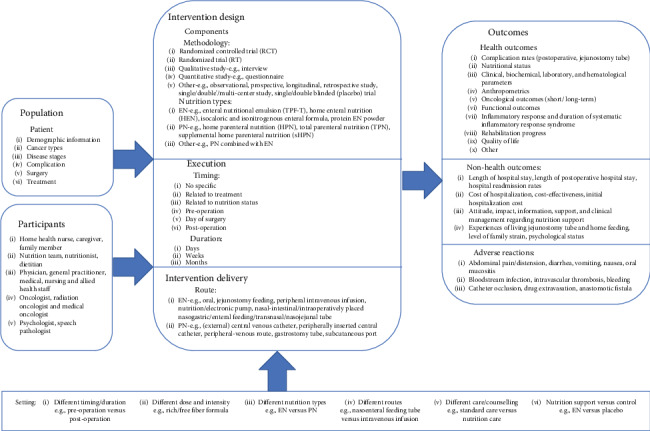
First version of the logic model.

The logic model included seven parts: population, participants, intervention design, execution, intervention delivery, outcomes, and setting (see Figure 1).

Population comprised patient's demographic information and medical information, such as disease stages and treatments. Participants included all the people involved in nutrition support for cancer patients.

Intervention design was divided into methodology and nutrition types. Execution included timing and duration of nutrition support. Many starting points were related to surgery, such as the day preoperative or postoperative. Intervention delivery was the routes of EN and PN.

Outcomes were divided to health outcomes, nonhealth outcomes, and adverse reaction. Nutrition support can affect various health outcomes, such as complication rates, nutritional parameters, anthropometrics, oncological outcomes, and quality of life. The nonhealth outcomes included length of hospital stay, cost of hospitalization, and the psychological status. However, there were some adverse reactions caused by nutrition support, such as diarrhea, vomiting, bloodstream infection, and anastomotic fistula.

Settings can be divided to different timing/duration (preoperation versus postoperation), dose and intensity (rich/free fiber formula), nutrition types (EN versus PN), routes (nasoenteral feeding tube versus intravenous infusion), care/counselling (standard care versus nutrition care), and nutrition support versus control (EN versus placebo).

### 3.3. Second Version of the Logic Model

**Figure 2:**
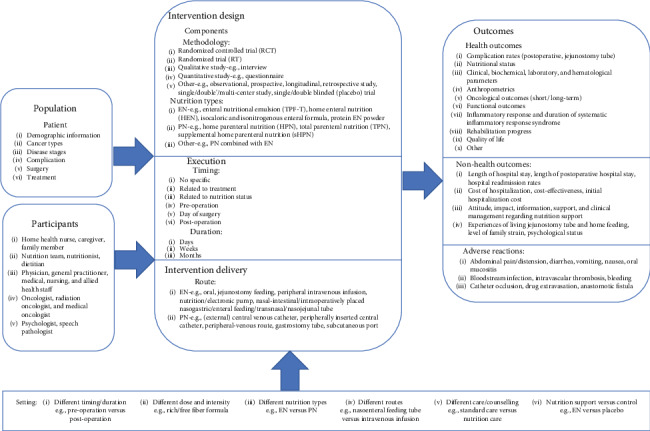
Second version of the logic model.

Based on the first version of the logic model, the second version of the logic model was developed after the interview. In the second versions of the logic model, the underlined elements in participants, nutrition types, timing, nutrition routes, health outcomes, nonhealth outcomes, and adverse reactions were emphasized by interviewees (see Figure 2).

In the second version of the logic model, participants including nutritionist, physician, family member, caregiver, and nursing and allied health staff were emphasized. EN was mainly given by oral, nasogastric tube, and nasojejunal tube, and PN was given via a central venous catheter and peripherally inserted central catheter. Many starting points of nutrition support were related to treatments and patient's nutrition status, and nutrition support is always accompanied with nutrition counselling.

For outcomes, clinical parameters, biochemical parameters, hematological parameters, rehabilitation progress, and anthropometrics, such as body weight and BMI, were the emphasized health outcomes. The nonhealth outcomes focused on the experiences and attitudes about nutrition support of cancer patients and length of hospital stay. The emphasized adverse reactions were abdominal pain, distension, diarrhea, vomiting, and nausea.

### 3.4. Barriers and Suggestions of Nutrition Support

According to the literature and interview, this pilot study summarized the barriers of nutrition support among cancer patients and gave some suggestions for different populations, including policy maker, hospital, medical staff, and researcher (see Tables 4 and 5).

One of the barriers of nutrition support in clinical practice is misunderstanding of the cancer patients and their families. Medical staff can refer cancer patients and their families to the nutritionists before receiving nutrition support so as to enhance their adherence to nutrition support. Nutrition support is always accompanied with long-term nutrition counselling for cancer patients and their families, and it takes lot of human resource and time which causes over workload. Hospitals should set up guidance to strengthen cooperation and communication between medical staff.

Economic factors, such as price and economic condition, are another barrier of nutrition support. The nutrition products involved in basic medical insurance drug catalogue should be regularly updated, and medical staff should try to understand the patient's financial situation and provide several nutrition support for cancer patients and their families.

## 4. Discussion

A system-based logic model of nutrition support for cancer patients was developed and validated by medical staff in this pilot study. The logic model was widely used to improve health problems. However, studies about the logic model of nutrition support for cancer patients were not conducted in the literature. The logic model was developed and validated in this study as it can serve as a tool to present a framework of nutrition support among cancer patients objectively and visually, and the elements involved in the logic model covered the main elements of nutrition support among cancer patients including seven parts: population, participants, intervention design, execution, intervention delivery, outcomes, and setting. This visual and descriptive model can function as a tool that facilitates communication and understanding between the different stakeholders in order to assist clinicians and hospital management in the process of nutrition support and standardize the implementation of nutrition support in clinical practice.

For this study, the first version of the logic model which was based on the current literature presented the basic elements of nutrition support for cancer patients. Furthermore, the second version of logic model was developed by combining the first version of the logic model and the interviews with medical staff in Macao. Comparatively, some elements in participants, nutrition types, timing, nutrition routes, health outcomes, nonhealth outcomes, and adverse reactions were added in the second version of the logic model. These elements reflected a more realistic operation of cancer nutrition care.

The elements emphasized in the second version of the logic model may be due to the human resource. The region with more medical resource and higher-level hospital owns adequate human resource which causes more medical staff to participate in the program of nutrition support and clear division of labor. For example, it is said that nurses were ideal to promote nutritional behaviors to cancer patients when dieticians or nutritionists were limited in Australia and New Zealand [56].

The types, routes, and timing of nutrition support emphasized may be caused by the gap between study and clinical practices. Only a limited number of EN and PN are widely used in clinical practice, and not every cancer patient undergoes surgery though many studies focus on the nutrition support for cancer patients with surgeries and conduct interventions with different timing related to surgery.

Health outcomes which were easy to be recorded, such as biochemical and hematological parameters, rehabilitation progress, and body weight, were emphasized by medical staff. The emphasized nonhealth outcomes focused on the experiences and attitudes about nutrition support of cancer patients, psychological status, and level of family strain because they can affect the patient's adherence to nutrition support. Adverse reactions caused by nutrition support were seldom observed solely, and it may be caused by other factors, such as cancer therapies and drugs.

There were also some barriers of nutrition support in practice. Based on the literature, economic factors [57], low adherence to guidelines [11, 58], and behavior of medical staff [15–17] are the barriers of nutrition support. In this study, medical staff in Macao mentioned other barriers of nutrition support, including misunderstanding of the cancer patients and their families, lack of standard guidelines, over workload, and loss of appetite of cancer patients. In addition, medical staff emphasized that the interaction between cancer patients and medical staff played an important role. Many cancer patients misunderstand the importance of nutrition support and even refuse to receive nutrition support. Thus, nutrition support is always accompanied with nutrition counselling for cancer patients and their families during the whole therapy.

Although there were guidelines of nutrition support for cancer patients [8–10], some medical staff lack related knowledge and there are no guidance or guidelines provided from hospitals. Hospitals should provide guidance and regular trainings for medical staff, especially opportunity for training out in order to communicate with professionals in different places and learn new technology.

The views of nutrition support have changed rapidly during the past decades [59]; policy makers and researchers should strengthen cooperation to standard utilization of nutrition support among cancer patients, such as organizing professionals to develop guidelines. For instance, ESPEN established terminology consensus for terminology of nutritional concepts and procedures as difference of opinion about terminology limits the development of clinical nutrition practice and research [60, 61]. On the other hand, researchers should consider how to transfer the research findings to clinical practice efficiently so as to narrow the gaps between theoretical studies and clinical practice in nutrition support. Furthermore, researches could try more applications for conference or workshop so as to improve the communication and cooperation between researches and institutions as the number of conference related to nutrition is limited. Only 4% of conferences grant applications related to nutrition awarded by the National Institutes of Health between 2000 and 2005, and the largest proportion of these awards belonged to the National Cancer Institute [62].

The logic model developed in this study can be adjusted to assist evaluating and designing interventions for improvement of cancer nutrition in different settings. In particular, based on this pilot study, there were some suggestions to use the logic model of nutrition support among cancer patients for future studies: (1) mind the difference between nutrition support and diet/dietary while literature search; (2) classify the literature based on the cancer types and disease stages; (3) make sure that interviewees have worked directly in the field of cancer and nutrition; (4) focus on the barriers and suggestions while interview; and (5) concentrate on the interaction between cancer patients and medical staff.

There were some limitations in this study. Firstly, this study only included literature from three databases during the past five years and did not include the grey literature. Secondly, only three medical staff in Macao participated in the interview in this pilot study.

## 5. Conclusion

The logic model was developed and validated in this pilot study, which provided a holistic framework of utilization of nutrition support among cancer patients for future research. The logic model can serve as a tool to better understand interactions between the intervention of nutrition support and its multiple outcomes among cancer patients.

## Figures and Tables

**Table 1:** PICO framework (population, intervention, comparison, and outcomes).

PICO	
Population	Which population are the target population and its information?
	Patients with cancer receiving nutrition support. Demographic information of cancer patients.
Intervention	What kind of intervention will be performed among cancer patients?
	Activities related to nutrition and performed among cancer patients and that caused specific outcomes.
Comparison	How to conduct comparison?
	Difference based on the actions or changes.
Outcomes	Which types of outcomes will be caused? Positive or negative?
	Changes that were caused by specific actions directly or indirectly, such as biochemical index, mortality, length of hospital stay, quality of life, and medical cost.

**Table 2:** Summary of study design of included literatures.

Design	Number	Papers
Randomized controlled trial (RCT)	5	Zhao et al. (2017), Obling et al. (2017), Cereda et al. (2018), Kabata et al. (2015), Hatao et al. (2017)
Randomized trial (RT)	6	Takesue et al. (2015), Okada et al. (2017), De Luis et al. (2015), Sánchez-Lara et al. (2014), Gavazzi et al. (2016), Vidal et al. (2016)
Longitudinal study	1	Vashi et al. (2014)
Observational study	1	Brown et al. (2017)
Interview	1	Cohen et al. (2017)
Questionnaire	1	Santarpia and Bozzetti (2018)
Cohort study	1	Shenep et al. (2017)
Single/double/multicenter study	1	Senesse et al. (2015)
Mixed method	13	Wang et al. (2015), Bowrey et al. (2015), Wang et al. (2018), Yu et al. (2017), Jin et al. (2018), Ding et al. (2015), Yang et al. (2015), Li et al. (2015), Cotogni et al. (2017), Kobayashi et al. (2017), Miyata et al. (2017), Li et al. (2015), Mendivil et al. (2017)
Total	30	

**Table 3:** Elements of included literatures.

Elements	Contents
Demographic information	(i) Gender
	(ii) Age
Cancer types	(i) (Incurable/upper/primary/secondary) gastrointestinal cancer, esophageal cancer, esophageal cancer, rectal carcinoma, colon carcinoma, duodenal carcinoma
	(ii) (Advanced-stage epithelial) ovarian cancer, fallopian tube and primary peritoneal cancer, pancreatic carcinoma, appendix cancer, head and neck cancer, advanced non-small-cell lung cancer, hepatic carcinoma, bladder cancer
	(iii) Abdominal cavity malignancy, cancer-related cachexia, pediatric oncology
Disease stages	(i) Stages I–IV, advanced stage
Surgery	(i) Thoracic surgery
	(ii) Elective esophagectomy, esophageal resection
	(iii) Total gastrectomy, radical gastrectomy
	(iv) Elective major gastrointestinal tract surgery, bowel resection
	(v) Ablative surgery, extended pelvic lymphadenectomy, radical cystectomy, cytoreductive surgery, debulking surgery
Treatments	(i) Chemotherapy (systemic, neoadjuvant), hormonal therapy, paclitaxel and cisplatin/carboplatin treatment, radiotherapy (plus systemic treatment), surgical therapy
Participants	(i) Patient, home health nurse, caregiver, physician, family member, nutrition team, nutritionist, general practitioner, oncologist, medical, nursing and allied health staff, dietitian, psychologist, speech pathologist, radiation oncologist, and medical oncologist.
Nutrition types	(i) EN: enteral nutritional emulsion (TPF-T), isocaloric and isonitrogenous enteral formula, protein EN powder, home enteral nutrition (HEN), omega-3-rich EN
	(ii) PN: home parenteral nutrition (HPN), total parenteral nutrition (TPN), supplemental home parenteral nutrition (sHPN)
	(iii) Other: fiber-free/enriched/and probiotic-enriched nutrition formula, low-nitrogen and low-calorie PN combined with EN
Nutrition routes	(i) EN: nutrition pump, jejunostomy feeding, oral, electronic pump, peripheral intravenous infusion, nasal-intestinal tube, intraoperatively placed nasogastric tube, enteral feeding tube, oral feeding, transnasal tube
	(ii) PN: central venous catheter, subcutaneous port, external central venous catheter, peripheral-venous route, gastrostomy tube, peripherally inserted central catheter
Timing	(i) No specific
	(ii) No more than 7 days prior to chemotherapy administration, after the first chemotherapy cycle, and after the second chemotherapy cycle, day 3 before the initiation of chemotherapy to day 12 of chemotherapy
	(iii) Preoperation: 1 day, 1 week
	(iv) Day of surgery
	(v) Postoperation: 24, 48, and 48-72 hours, 7 days, the day that the patient began eating a postoperative diet
Duration	(i) Days: 2-14
	(ii) Weeks: 1-6
	(iii) Months: 2-6
Health outcomes	(i) Complication rates (postoperative, jejunostomy tube)
	(ii) Nutritional status nutritional assessment
	(iii) Clinical, biochemical, laboratory, and hematological parameters, blood chemistry
	(iv) Anthropometrics, e.g., body weight, body composition, rate of weight loss, fat free mass index, fat free mass, handgrip strength, muscle strength, six-minute walking test, protein-calorie intake, and caloric intake
	(v) Oncological outcomes (short-/long-term), e.g., chemotherapy-related toxicities, response to chemotherapy and survival, and anticancer treatment tolerance
	(vi) Functional outcomes, e.g., functional status (Karnofsky performance status (KPS)), immune function, liver function indexes, intestinal function recovery, functional capacity, bowel movement recovery, and restoration of bowel function
	(vii) Inflammatory markers, such as tumor necrosis factor- (TNF-) a and interleukin- (IL-) 6, immunoglobulins, CD3+, CD4+, CD8+, and natural killer cells, albumin and prealbumin, hemoglobin, inflammatory response, and duration of systematic inflammatory response syndrome
	(viii) Quality of life (generic and disease-specific), e.g., physical/role/emotional functioning, appetite loss, and fatigue
	(ix) Other, e.g., days for first fecal passage, blood glucose (BG) values, adherence to nutrition support, and phase angle
Nonhealth outcomes	(i) Length of hospital stay, length of postoperative hospital stay, hospital readmission rates
	(ii) Cost of hospitalization, cost-effectiveness, initial hospitalization cost
	(iii) Attitude, impact, information, support, and clinical management regarding nutrition support
	(iv) Experiences of living jejunostomy tube and home feeding, level of family strain, psychological status
Adverse reactions	(i) Abdominal pain, vomiting/nausea, anastomotic fistula, and abdominal distension, oral mucositis, diarrhea, bloodstream infection, catheter occlusion, drug extravasation, intravascular thrombosis, bleeding, exudates, swelling, induration
Setting	(i) Different timing/duration (preoperation versus postoperation)
	(ii) Different dose and intensity (rich/free fiber formula)
	(iii) Different nutrition types (EN versus PN)
	(iv) Different routes (nasoenteral feeding tube versus intravenous infusion)
	(v) Different care/counselling (standard care versus nutrition care)
	(vi) Different nutrition support versus control (EN versus placebo)

**Table 4:** Barriers of nutrition support among cancer patients.

Barriers	Possible reason
Misunderstanding of nutrition support	Many cancer patients and their families ignored the importance of nutrition support and misunderstand its effects.
Economic factors	Cancer therapies are long-term and costly. The nutrition products covered by social insurance are a priority to use in clinical practice, and the number of EN products covered by social insurance is less than that of PN products [57].
Lack of standard guidelines	The adherence to the guidelines published by nutritional institutions needs to be improved, and the medical staff want more detailed and practical guidance.
Over workload	Nutrition support always accompanies with long-term nutrition counselling for cancer patients during the whole therapy program, and it takes lot of human resource.
Loss of appetite	Many cancer patients will lose their appetites and need regular nutrition assessment and adjustment of nutrition support.

**Table 5:** Suggestions for different populations.

Population	Suggestions
Policy maker	To update nutrition product in basic medical insurance drug catalogue regularly. To organize professionals to develop guidelines for standard utilization of nutrition support among cancer patients.
Hospital	To provide regular trainings and consistent and useful guidance. To set up guidance to strengthen cooperation and communication between medical staff and introduce more nutritional supplements from foreign hospitals.
Medical staff	To refer cancer patients and their families to nutritionists before utilization of nutrition support. To discuss and provide several nutrition support for cancer patients.
Researchers	To consider how to transfer the research findings to clinical practice efficiently. To try more applications for conferences or workshops. To improve the communication and cooperation between researchers and institutions.

## Data Availability

All the data used are included in Results of this manuscript.
